# New Spectrophotometric and Fluorimetric Methods for Determination of Fluoxetine in Pharmaceutical Formulations

**DOI:** 10.1155/2009/257306

**Published:** 2009-08-16

**Authors:** Ibrahim A. Darwish, Sawsan M. Amer, Heba H. Abdine, Lama I. Al-Rayes

**Affiliations:** Department of Pharmaceutical Chemistry, College of Pharmacy, King Saud University, P.O. Box 2457, Riyadh 11451, Saudi Arabia

## Abstract

New simple and sensitive spectrophotometric and fluorimetric methods have been developed and validated for the determination of fluoxetine hydrochloride (FLX) in its pharmaceutical formulations. The spectrophotometric method was based on the reaction of FLX with 1,2-naphthoquinone-4-sulphonate (NQS) in an alkaline medium (pH 11) to form an orange-colored product that was measured at 490 nm. The fluorimetric method was based on the reaction of FLX with 4-chloro-7-nitrobenzo-2-oxa-1,3-diazole (NBD-Cl) in an alkaline medium (pH 8) to form a highly fluorescent product that was measured at 545 nm after excitation at 490 nm. The variables affecting the reactions of FLX with both NQS and NBD-Cl were carefully studied and optimized. The kinetics of the reactions were investigated, and the reaction mechanisms were presented. Under the optimum reaction conditions, good linear relationships were found between the readings and the concentrations of FLX in the ranges of 0.3–6 and 0.035–0.5 *μ*g mL^−1^ for the spectrophotometric and fluorimetric methods, respectively. The limits of detection were 0.1 and 0.01 *μ*g mL^−1^ for the spectrophotometric and fluorimetric methods, respectively. Both methods were successfully applied to the determination of FLX in its pharmaceutical formulations.

## 1. Introduction

Fluoxetine (FLX); (3*RS* **)-**N-methyl-3-phenyl-3-[4-(trifluoromethyl)phenoxy] propane-1-amine hydrochloride is the most widely prescribed selective serotonin reuptake inhibitor antidepressant drug [1]. FLX is also uniquely effective in treatment of the obsessive-compulsive disorders [2]. It is well absorbed after oral administration, and it takes 6–8 hours to reach the plasma peak. It has a long half-life time that allowed for the introduction of once-weekly dosing. The resolution of the adverse effects after discontinuation of FLX is slow [3]. These combined qualities made FLX the most widely prescribed antidepressant drug worldwide. 

 FLX has been determined in its pharmaceutical formulations by titrimetry [4], nuclear magnetic resonance spectrometry [5], potentiometry [6], thin-layer chromatography [7], liquid chromatography [4, 7], gas chromatography [8], and capillary electrophoresis [9]. These methods were time-consuming, tedious, and/or dedicated to sophisticated and expensive analytical instruments. Spectrophotometry [10–12] and fluorimetry [13–15] are the most convenient techniques because of their inherent simplicity, high sensitivity, low cost, and wide availability in quality control laboratories. Unfortunately, the spectrophotometric [16–24] and fluorimetric [25–27] methods that have been reported for determination of FLX in its pharmaceutical formulations were associated with some major drawbacks such as the lack of selectivity [16], tedious extraction procedures [20–24], and time-consuming [17, 26]. Therefore, the development of new alternative spectrophotometric and fluorimetric methods for the determination of FLX that can overcome the drawbacks of the existing methods was very essential. 

 1,2-Naphthoquinone-4-sulphonate (NQS) [28–30] and 4-chloro-7-nitrobenzo-2-oxa-1,3-diazole (NBD-Cl) [31–33] have been used as derivatizing reagents in the development of both spectrophotometric and fluorimetric methods for determination of many pharmaceutical amines. FLX, being amine, is a potential candidate for the reaction with both NQS and NBD-Cl. The present study was devoted to investigate the reaction of FLX with both reagents, and employment the reactions in the development of new simple spectrophotometric and fluorimetric methods for the determination of FLX in its pharmaceutical formulations.

## 2. Experimental

### 2.1. Apparatus

Double beam V-530 (JASCO Co. Ltd., Kyoto, Japan) ultraviolet-visible spectrophotometer with matched 1-cm quartz cells was used for all the spectrophotometric measurements. FP-6200 fluorometer (JASCO Co. Ltd.), with 1-cm quartz cells, was used for the fluorimetric measurements. Also, pH meter, Model 350 (Bibby Scientific Ltd., T/As Jenway, Essex, England) and MLW type thermostatically controlled water bath (Memmert GmbH, Co. Schwa bach, Germany) were used.

### 2.2. Reagents and Materials

Fluoxetine hydrochloride (FLX; Solvay Pharma, Suresnes, France) was obtained and used as received; its purity was 99.5 ± 1.55%. A solution of 0.5% (w/v) of 1,2-naphthoquinone-4-sulphonate (NQS; Aldrich Chemical Co., St. Louis, USA) was prepared by dissolving 250 mg in 50 mL distilled water. The solution was freshly prepared and protected from light during use. A solution of 0.2% (w/v) of 4-chloro-7-nitrobenzo-2-oxa-1,3-diazole (NBD-Cl; Sigma Chemical Co., St. Louis, USA) was freshly prepared by dissolving 100 mg in 50 mL acetone. Clark and Lubs buffer solution was prepared by mixing 50 mL of 0.2 M aqueous solution of boric acid and potassium chloride (1 liter contains 12.368 g of boric acid and 14.90 g of potassium chloride) with 21.3 mL of 0.2 M sodium hydroxide in 200 mL standard flask [34], and adjusted by pH meter. The following pharmaceutical formulations were used: prozac (Eli Lilly & Co. Ltd., Hampshire, UK), Fluzac (Riyadh Pharma, Riyadh, Saudi Arabia), salipax (Mepha Ltd. Aesch-Basilea Switzerland), flutin (Egyptian International Pharmaceutical Industries Co., Cairo, Egypt), and octozac (October Pharma, Cairo, Egypt) capsules are labeled to contain 20 mg FLX per capsule. Double distilled water was obtained through WSC-85 water purification system (Hamilton Laboratory Glass Ltd., Kent, USA) and used throughout the work. All solvents and materials used throughout this study were of analytical grade.

### 2.3. Preparation of Solutions

#### 2.3.1. Standard FLX Solution

An accurately weighed amount (50 mg) of FLX was quantitatively transferred into a 25-mL calibrated flask, dissolved in 20 mL distilled water, completed to volume with the same solvent to obtain a stock solution of 2 mg mL^−1^. The stock solution was found to be stable for at least two weeks when kept in refrigerator. The stock solution was further diluted with water to obtain working solutions in the range of 3–60 *μ*g mL^−1^ and 0.35–5 *μ*g mL^−1^ for the spectrophotometric and fluorimetric methods, respectively.

#### 2.3.2. Pharmaceutical Formulation Samples

The contents of 20 capsules were weighed, and finely powdered. An accurately weighed quantity of the powder equivalent to 100 mg of the active ingredient was transferred into a 100-mL calibrated flask, and dissolved in about 40 mL of distilled water. The contents of the flask were swirled, sonicated for 5 minutes, and then completed to volume with water. The contents were mixed well and filtered; the first portion of the filtrate was rejected. The filtered solution was diluted quantitatively with distilled water to obtain suitable concentrations for the analysis by the spectrophotometric and fluorimetric methods.

### 2.4. General Recommended Procedures

#### 2.4.1. Spectrophotometric Method

One milliliter of FLX solution containing 3–60 *μ*g mL^−1^ was transferred into separate 10-mL calibrated flask. One milliliter of Clark and Lubs buffer solution of pH 11 was added followed by 1 mL of NQS solution (0.5%, w/v). The reaction solution was allowed to proceed at room temperature (25 ± 5°C) for 10 minutes, and completed to volume with methanol. The resulting solution was measured at 490 nm against reagent blank prepared in the same manner with 1 mL water instead of 1 mL sample solution.

#### 2.4.2. Fluorimetric Method

One milliliter of FLX solution containing 0.35–5 *μ*g mL^−1^ was transferred into separate 10-mL calibrated flasks. One milliliter of Clark and Lubs buffer solution of pH 8 ± 0.2 was added followed by 1 mL of 0.2% (w/v) NBD-Cl solution. The reaction mixture was allowed to proceed in thermostatically controlled water bath at 70°C for 20 minutes, and then cooled to room temperature (25 ± 5°C). After cooling, the reaction mixture was acidified by adding 1 mL of 0.1 M, and completed to volume with acetonitrile. The relative fluorescence intensity (RFI) of the resulting solution was measured at *λ*_ex_ = 490 nm, *λ*_em_ = 545 nm against reagent blank prepared in the same manner with 1 mL water instead of 1 mL sample solution.

### 2.5. Determination of Stoichiometric Ratio

The limiting logarithmic method [35] was employed. Two sets of experiments were carried out employing the general recommended procedures described above. The first set of experiments was carried using varying concentrations of the analytical reagent with a fixed concentration of FLX. The second set of experiments was carried using varying concentrations of FLX at a fixed concentration of each reagent. The logarithms of the obtained absorbances (for the reaction with NQS) and RFI (for the reaction with NBD-Cl) were plotted as a function of the logarithms of the concentrations of the reagent and FLX in the first and second sets of experiments, respectively. The slopes of the fitting lines in both sets of experiments were calculated. The concentrations used in these experiments were as follows.

#### 2.5.1. Reaction with NQS

The first set of experiments was carried using varying concentrations of NQS (1.92 × 10^−4^–9.61 × 10^−4^ M) at fixed concentration of FLX (1.16 × 10^−5^ M). The second set of experiments was carried using varying concentrations of FLX (4.60 × 10^−7^–1.53 × 10^−5^ M) at a fixed concentration of NQS (1.92 × 10^−3^ M).

#### 2.5.2. Reaction with NBD-Cl

The first set of experiments was carried using varying concentrations of NBD-Cl (5 × 10^−5^–5 × 10^−4^ M) at fixed concentration of FLX (5.78 × 10^−7^ M). The second set of experiments was carried using varying concentrations of FLX (5.78 × 10^−8^–6.94 × 10^−7^ M) at a fixed concentration of NBD-Cl (1 × 10^−3^ M).

## 3. Results and Discussion

### 3.1. Absorption and Fluorescence Spectra

**Figure 1:**
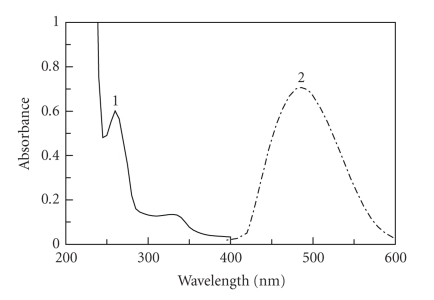
Absorption spectra of FLX (100 *μ*g mL^−1^) against water (1) and its reaction product with NQS (0.5%, w/v) against reagent blank (2).

The absorption spectrum of FLX was recorded against water (Figure 1). It was found that the maximum absorption peak (*λ*_max _) of FLX was 260 nm, and its molar absorptivity (*ε*) was 1.3 × 10^4^ l mol^−1^ cm^−1^. Because of the highly blue shifted *λ*_max_ of FLX, its determination in the pharmaceutical formulations based on the direct measurement of its absorption for ultraviolet light is susceptible to potential interferences from the coextracted common excipients. As well, the low *ε* value could ultimately result in poor sensitivity. Therefore, derivatization of FLX to more red-shifted light-absorbing derivative was necessary. The reaction between FLX and NQS was performed, and the absorption spectrum of the product was recorded against reagent blank. The product was orange-colored exhibiting *λ*_max_ at 490 nm (Figure 1). Obviously, the *λ*_max_ of FLX-NQS derivative was red-shifted by 230 nm. This high red shift could ultimately eliminate any potential interference. As well, the value of *ε* (sensitivity) was greatly enhanced to be 4.8 × 10^4^ l mol^−1^ cm^−1^. Therefore, the spectrophotometric measurements were carried out at 490 nm. 

**Figure 2:**
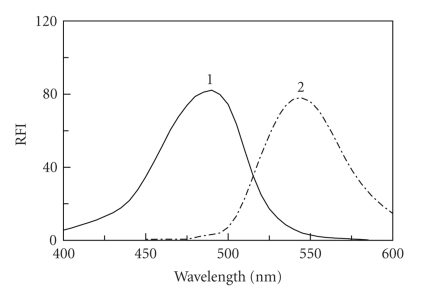
Excitation (1) and emission (2) spectra of FLX (0.03 *μ*g mL^−1^) and its reaction product with NBD-Cl (0.2%, w/v) against reagent blank.

 FLX does not have a native fluorescence, thus its derivatization with fluorogenic reagent was necessary for its fluorimetric determination. NBD-Cl forms highly fluorescent derivative with secondary amines using relatively mild reaction conditions [31–33]; therefore, it was chosen as a derivatizing reagent for FLX. Owing to the presence of labile chloride in the chemical structure of NBD-Cl, a daily fresh solution was prepared and tested in the present study. It was found that FLX reacts with NBD-Cl and forms yellow-colored fluorescent derivative. This derivative exhibited maximum fluorescence intensity (*λ*_em_) at 545 nm after its excitation at wavelength (*λ*_ex_) of 490 nm. The excitation and emission spectra for the reaction product of FLX with NBD-Cl are given in Figure 2.

### 3.2. Optimization of Reaction Variables

#### 3.2.1. Effect of Reagent Concentration

**Figure 3:**
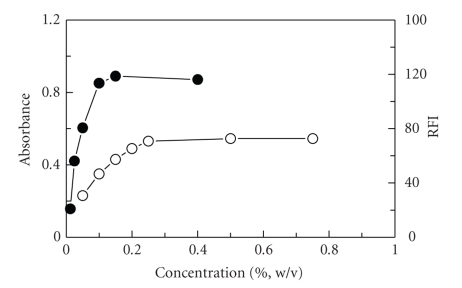
Effect of NQS (•) and NBD-Cl (*∘*) concentrations on their reaction with FLX. FLX concentrations were 3 and 0.02 *μ*g mL^−1^ for its reaction with NQS and NBD-Cl, respectively. RFI is the relative fluorescence intensity.

The effect of NQS and NBD-Cl concentrations on their reactions with FLX revealed that the reactions were dependent on the reagent concentration as the readings increased with the increase in the reagent concentration (Figure 3). The highest readings were attained at concentration ranges of 0.25–1 and 0.1–0.4% (w/v) for NQS and NBD-Cl, respectively. For high precise values, further experiments were carried out using 0.5 and 0.2% for NQS and NBD-Cl, respectively.

#### 3.2.2. Effect of pH

**Figure 4:**
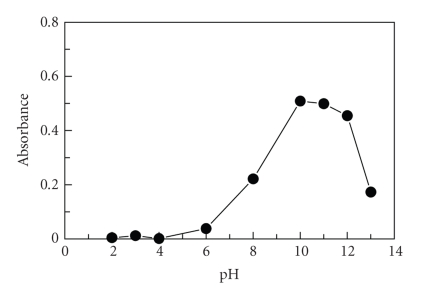
Effect of pH on the reaction of FLX with NQS (0.5%, w/v) and NBD-Cl (0.2%, w/v). FLX concentrations were 3 and 0.02 *μ*g mL^−1^ for its reaction with NQS and NBD-Cl, respectively. RFI is the relative fluorescence intensity.

The influence of pH on the reaction of FLX with both NQS and NBD-Cl was investigated by carrying out the reaction in buffer solution of varying pH values. The results revealed that FLX has difficulty to react with both NQS and NBD-Cl in acidic media (Figure 4). This was possibly due to the existence of the amino group of FLX in the form of hydrochloride salt, thus it loses its nucleophilic substitution capability. As the pH increased, the readings increased rapidly, as the amino group of FLX (in the hydrochloride salt) turns into the free amino group, thus facilitating the nucleophilic substitution. The maximum readings were attained at pH values of 11 and 8 for the reaction with NQS and NBD-Cl, respectively. At higher pH values, sharp decrease in the readings occurred. This was attributed probably to the increase in the amount of hydroxide ion that holds back the reaction of FLX with NQS and NBD-Cl.

#### 3.2.3. Effect of Temperature and Time

**Figure 5:**
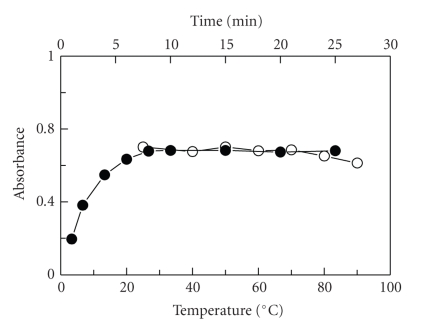
Effect of temperature (•) and time (*∘*) on the reaction of FLX (3 *μ*g mL^−1^) with NQS (0.5%, w/v).

The effect of temperature on the reaction was studied by carrying out the reaction at different temperatures (25–90°C). It was found that the reaction of FLX with NQS was not affected by increasing the temperature, and the reaction at room temperature (25 ± 5°C) went to completion in 10 minutes, and longer reaction time up to 25 minutes did not affect the reaction (Figure 5). Therefore, further experiments involving NQS reagent were carried out at room temperature (25 ± 5°C) for 10 minutes. 

**Figure 6:**
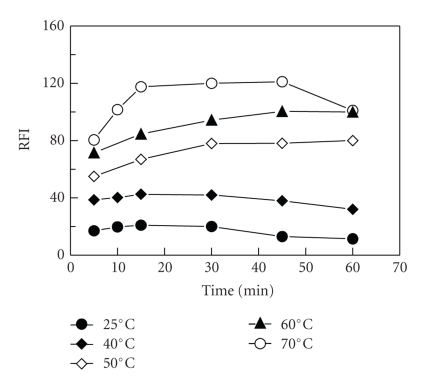
Effect of time on the reaction of FLX (0.02 *μ*g mL^−1^) with NBD-Cl (0.2%, w/v) at different temperatures. RFI is the relative fluorescence intensity.

 The reaction of FLX with NBD-Cl at room temperature (25 ± 5°C) was found to be very slow, as it required more than 1 hour for completion (Figure 6). Therefore, the reaction was carried out at varying elevated temperatures (40–70°C) for varying times, and the intensities of the induced fluorescence were measured. The optimum conditions were considered as the conditions at which high RFI values, high reproducible results, and comfortable measurements (wide plateau region on the RFI-time curve) could be obtained. The results indicated that the reaction was dependent on temperature, and the optimum conditions were achieved by heating the reaction at 70°C for 20 minutes (Figure 6).

#### 3.2.4. Effect of Diluting Solvent

Upon diluting the reaction solutions with water, colloids were obtained indicating the incomplete solubility of FLX-NQS and FLX-NBD derivatives in water. Therefore, water could not be used for dilution. In order to select the most appropriate organic solvent for diluting the reaction solutions, different solvents were tested: methanol, ethanol, isopropanol, acetone, acetonitrile, dimethylsulphoxide, and 1,4-dioxane. The highest readings were obtained when methanol was used for dilution. With NBD-Cl, significantly high fluorescence background was observed. This was attributed to the hydrolysis of NBD-Cl to the corresponding hydroxy derivative, namely, 4-hydroxy-7-nitrobenzo-2-oxa-1,3-diazole (NBD-OH) [36]. The fluorescence of NBD-OH was found to be quenched by decreasing the pH of the reaction medium to less than one [37]. Therefore acidification of the reaction mixture prior to dilution and measurement of the RFI was necessary to remarkably decrease the background fluorescence. Meanwhile, the reaction product was not affected, thus the sensitivity was ultimately enhanced. It was found that the concentration of HCl required for acidification was 0.01 M in the final assay solutions (i.e., 1 mL of 0.1 M).

#### 3.2.5. Stability of the Chromophore and Fluorophore

After dilution the reaction solutions, it was found that the absorbance of the chromogen (FLX-NQS) and the FI of the fluorophore (FLX-NBD) remained stable for at least 4 hours. This allowed the processing of large batches of samples, and their comfortable measurements with convenience. This increased the convenience of the methods as well as made the method applicable for large number of samples.

### 3.3. Stoichiometry and Kinetics of the Reactions

**Figure 7:**
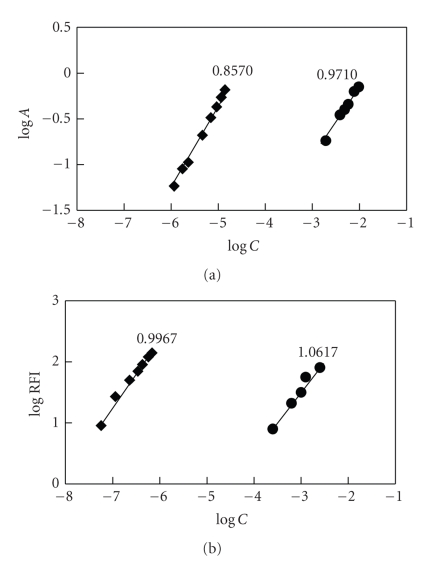
Limiting logarithmic plots for molar reactivity of FLX with NQS (a) and NBD-Cl (b). *C, A,* and RFI are the concentration, absorbance, and relative fluorescence intensity, respectively.

**Figure 8:**
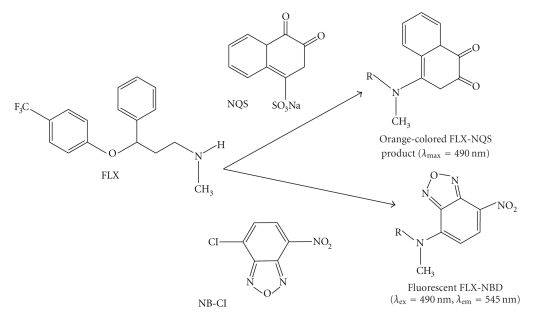
Scheme for the reaction pathway of FLX with NQS and NBD-Cl.

Under the optimum conditions (Table 1), the stoichiometries of the reaction of FLX with both NQS and NBD-Cl were investigated by the limiting logarithmic method [35]. In each case, two straight lines were obtained (Figure 7). The slopes of these lines were comparable confirming the 1 : 1 ratio for the reactions. Based on this ratio, the reaction pathways were postulated to be proceeded as shown in Figure 8. 

 Under the optimum conditions, the signal-time curves for the reactions at varying concentrations of FLX (1.1 × 10^−6^–1.6 × 10^−5^ and 2.3 × 10^−6^–9.2 × 10^−6^ M for the reactions with NQS and NBD-Cl, resp.) with a fixed concentration of NQS (1.92 × 10^−3^ M) and NBD-Cl (1 × 10^−3^ M) were generated. The initial reaction rates (*K*) were determined from the slopes of these curves. The logarithms of the reaction rates (Log *K*) were plotted as a function of logarithms of FLX concentrations (log  *C*). Straight lines with slope values of 0.9708 (with NQS) and 0.8378 (with NBD-Cl) were obtained by fitting the data to the following equation:

(1) $$\text{Log}\;K=\log\;\;K^{\prime }+\;n\;\log\;\;C,$$

where *K * is reaction rate, *K*′ is the rate constant, *C * is the molar concentration of FLX, and *n * (slope of regression line) is the order of the reaction. The values of the slopes (≈1) confirmed that the reactions were first order. However under the optimized reaction conditions, the concentrations of NQS and NBD-Cl were in much more excess than that of FLX in the reaction solution. Therefore, the reactions were regarded as pseudo-first order reactions.

### 3.4. The Apparent Rate Constant and Activation Energy

**Figure 9:**
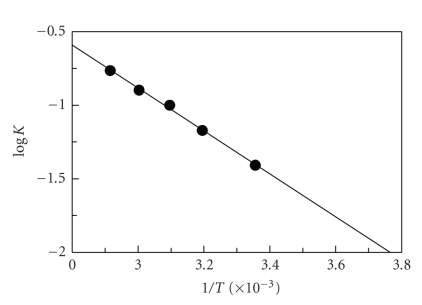
Arrhenius plot for the reaction of FLX with NBD-Cl. *T * and *K * are the absolute temperature and the apparent rate constant, respectively. (FLX) and (NBD-Cl) were 4.3 × 10^−7^ and 1 × 10^−3^ M, respectively.

As the reaction of FLX with NQS was not affected by the temperature, therefore, it was ruled out from this investigation. The RFI-time curves for the reaction of FLX with NBD-Cl were generated by carrying out the reaction at different temperatures (25, 40, 50, 60, and 70°C) using fixed concentration of FLX (4.3 × 10^−7^ M), and NBD-Cl (1 × 10^−3^ M). From these curves, the apparent rate constant was calculated. The activation energy, defined as the minimum kinetic energy that a molecule possess in order to undergo a reaction, was determined using Arrhenius equation [38]:

(2) $$\text{Log}\;k=\log\;\;A-\frac{\text{Ea}}{2.303}\;RT,$$

where *k * is the apparent rate constant, *A * is the frequency factor, Ea is the activation energy, *T * is the absolute temperature, and *R * is the gas constant (1.987 cal degree^−1^ mole^−1^). The values of log  *k* were plotted as a function of 1/*T*. Straight line with slope (= − Ea/2.303* R*) values of −1.4598 was obtained (Figure 9). The activation energy was calculated and found to be 6.68 kcal mole^−1^. This low activation energy explains that the reaction of FLX with NBD-Cl could easily take place under mild conditions, and NBD-Cl could be used as a useful analytical reagent in the fluorimetric determination of FLX. 

### 3.5. Validation of the Methods

#### 3.5.1. Linearity, Limits of Detection

In the proposed methods, linear plots (*n* = 5) with good correlation coefficients were obtained in the concentration ranges of 0.3–6 and 0.035–0.5 *μ*g mL^−1^ for the spectrophotometric and fluorometric methods, respectively (Table 2). The limits of detection (LOD) and quantitation (LOQ) were determined [39] using the formula: LOD or LOQ = *κ*SDa/b, where *κ * = 3.3 for LOD and 10 for LOQ, SDa is the standard deviation of the intercept, and b is the slope. The LOD values were 0.1 and 0.011 *μ*g mL^−1^ and LOQ were 0.3 and 0.033 *μ*g mL^−1^, for the spectrophotometric and fluorimetric methods, respectively (Table 2).

#### 3.5.2. Reproducibility

The reproducibility of the proposed methods was determined by replicate analysis of five separate solutions of the working standards. The methods gave satisfactory results; the relative standard deviations (RSDs) were 1.52 and 3.37% for the spectrophotometric and fluorimetric methods, respectively (Table 3), indicating good reproducibility of the proposed methods. This precision level is adequate for the precision and routine analysis of FLX in quality control laboratories.

#### 3.5.3. Accuracy and Specificity

The accuracy of the proposed methods was evaluated by the recovery studies for added concentrations of FLX. The recovery values were 98.8 ± 0.6–102.0 ± 1.0 and 97.5 ± 2.1–100.2 ± 2.2% for the spectrophotometric and fluorimetric methods, respectively (Table 4), indicating the accuracy of the proposed methods. The specificity of the methods was evaluated by investigating the interference liabilities from the common excipients that might be added during pharmaceutical formulation. Samples were prepared by mixing known amount (20 mg) of FLX with various amounts of the common excipients: starch, glucose, lactose, acacia, talc, and magnesium stearate. These laboratory-prepared samples were analyzed by the proposed methods applying the general recommended procedure. The average recovery values were 99.9 ± 0.4 and 99.1 ± 1.3% for the spectrophotometric and fluorimetric methods, respectively (Table 5). These data confirmed the absence of interference from any of the common excipients with the determination of FLX by both methods.

#### 3.5.4. Robustness and Ruggedness

Robustness was examined by evaluating the influence of small variation in the method variables on its analytical performance. In these experiments, one parameter was changed whereas the others were kept unchanged, and the recovery percentage was calculated each time. It was found that small variation in the method variables did not significantly affect the procedures; recovery values were 97.5–100.5 ± 1.3–1.9%. The most critical factor affecting the results was the pH that should be adjusted to be in the range of optimum ±0.2. Ruggedness was also tested by applying the methods to the assay of FLX using the same operational conditions but using two different instruments at two different laboratories and different elapsed time. Results obtained from lab-to-lab and day-to-day variations were reproducible, as the RSD did not exceed 4%.

### 3.6. Applications of the Methods

It is evident from the above-mentioned results that the proposed methods gave satisfactory results with FLX in its bulk. Thus its pharmaceutical formulations were subjected to the analysis of their FLX contents by the proposed and the official [16] methods. The label claim percentages were 99.8 ± 1.2–100.1 ± 1.6 and 99.2 ± 1.5–100.8 ± 1.8% for the spectrophotometric and fluorimetric methods, respectively (Table 6). These results were compared with those obtained from the official method by statistical analysis with respect to the accuracy (by *t*-test) and precision (by *F*-test). No significant differences were found between the calculated and theoretical values of *t*- and *F*-tests at 95% confidence level proving similar accuracy and precision in the determination of FLX by both methods.

## 4. Conclusions

The present paper described the evaluation of NQS and NBD-Cl as analytical reagents in the development of simple, sensitive, and accurate spectrophotometric and fluorimetric methods, respectively, for the determination of FLX in bulk and pharmaceutical formulations. The described methods are superior to the previously reported spectrophotometric or fluorimetric methods in terms of the simplicity and sensitivity. The proposed methods have comparable analytical performances and devoid from any potential interference. This gives the advantage of flexibility in performing the analysis on any available instrument. Therefore, these methods can be recommended for the routine analysis of FLX in quality control and clinical laboratories.

## Figures and Tables

**Table 1:** Summary for the optimization of variables affecting the reaction of FLX with NQS and NBD-Cl reagents employed in the development of the proposed spectrophotometric and fluorimetric methods, respectively.

Variable	Spectrophotometric method		Fluorimetric method	
	Studied range	Optimum	Studied range	Optimum
NQS (%, w/v)	0.05–0.8	0.5		
NBD-Cl (%, w/v)			0.01–0.5	0.2
pH	6–13	11	5–9.5	8 ± 0.2
Temperature (°C)	25–90	25	25–70	70
Time (minutes)	2–25	10	5–60	20
Diluting solvent	Different^(a)^	Methanol	Different^(a)^	Methanol^(b)^
Measuring wavelength (nm)	400–600	490	400–600	490 (*λ*_ex_), 545 (*λ*_em_)

^(a)^Solvents tested: methanol, ethanol, isopropanol, acetone, acetonitrile, dimethylsulphoxide, and 1,4-dioxane. ^(b)^One milliliter of 0.1 M HCl was added before dilution with methanol.

**Table 2:** Analytical parameters for the proposed spectrophotometric and fluorimetric methods for determination of FLX based on its reaction with NQS and NBD-Cl, respectively.

Parameter	Spectrophotometric	Fluorimetric
	method	method
Linear range (*μ*g mL^−1^)	0.3–6	0.035–0.5
Intercept	0.0006	2.1766
SD of intercept	0.0038	1.9249
Slope	0.1356	0.5793
SD of slope	0.0014	0.0084
Correlation coefficient	0.9997	0.9992
LOD (*μ*g mL^−1^)	0.1	0.011
LOQ ((*μ*g mL^−1^)	0.3	0.033

**Table 3:** Replicate analysis of FLX solution by the proposed spectrophotometric and fluorimetric methods.

Sample number	Absorbance	RFI
	(FLX = 3 *μ*g mL^−1^)	(FLX = 0.2 *μ*g mL^−1^)
1	0.453	120
2	0.458	110
3	0.451	117
4	0.471	115
5	0.459	121
Mean	0.458	116.6
SD	0.007	3.93
RSD	1.52	3.37

**Table 4:** Recovery studies for determination of FLX by the proposed spectrophotometric and fluorimetric methods.

Spectrophotometric method		Fluorimetric method	
Added (*μ*g mL^−1^)	Recovery (% ± SD)^(a)^	Added (*μ*g mL^−1^)	Recovery (% ± SD)^(a)^
2.0	98.8 ± 0.6	0.10	97.5 ± 2.1
3.0	99.5 ± 1.0	0.15	98.1 ± 1.9
4.0	101.5 ± 1.2	0.20	100.2 ± 2.2
5.0	102.0 ± 1.0	0.25	99.6 ± 1.5
6.0	99.0 ± 0.8	0.30	100.2 ± 1.9

^(a)^Values are mean of three determinations.

**Table 5:** Analysis of FLX in presence of common excipients by the proposed spectrophotometric and fluorimetric methods.

Excipient		
	Recovery (% ± SD)^(a)^	
	Spectrophotometric	Fluorimetric
	method	method
Starch (50)^(b)^	99.6 ± 0.8	101.0 ± 2.2
Glucose (10)	100.1 ± 1.0	98.7 ± 2.1
Lactose (10)	99.8 ± 0.5	99.6 ± 1.9
Acacia (10)	100.2 ± 0.9	98.7 ± 1.4
Talc (5)	99.6 ± 1.0	99.4 ± 1.3
MS^(c)^ (10)	100.1 ± 0.1	97.0 ± 2.8
Average ± SD	99.9 ± 0.4	99.1 ± 1.3

^(a)^Values are mean of three determinations. ^(b)^Figures in parenthesis are the amounts in mg added to 20 mg of FLX. ^(c)^MS = Magnesium stearate.

**Table 6:** Determination of FLX in its pharmaceutical formulations (capsules) by the proposed spectrophotometric, fluorimetric and the official methods.

Capsules	Spectrophotometric method			Fluorimetric method		
	Recovery (%) ± SD^(a)^	*F * value^(b)^	*t * value^(b)^	Recovery (%) ± SD^(a)^	*F * value^(b)^	*t * value^(b)^
Prozac	99.9 ± 1.4	1.68	1.17	99.7 ± 1.6	2.38	3.00
Pluzac	100.1 ± 1.3	2.62	1.27	99.2 ± 1.5	1.58	2.91
Salipax	99.8 ± 1.3	1.00	1.10	100.8 ± 1.8	1.60	2.18
Flutin	100.1 ± 1.6	1.64	1.27	100.1 ± 1.3	0.19	1.12
Octozac	99.8 ± 1.2	2.35	2.13	99.4 ± 1.5	1.49	2.47

^(a)^Values are mean of five determinations ± SD. ^(b)^Theoretical values for *t * and *F * at 95% confidence limit (*n* = 5) were 2.78 and 6.39, respectively.
